# FreqMamba-Net: a frequency-enhanced state space model for fine-grained mucosal lesion segmentation in colonoscopy

**DOI:** 10.3389/fonc.2026.1847281

**Published:** 2026-07-22

**Authors:** Pengliang Zhang, Shuang Chen, Xianmin Liu, Lijuan Wu, Yingjian Zhang

**Affiliations:** 1 First Affiliated Hospital of Henan University of Science and Technology, Department of Gastroenterology, Luoyang, Henan, China; 2 First Affiliated Hospital of Henan University of Science and Technology, Cosmetic Medicine Center, Luoyang, Henan, China

**Keywords:** colonoscopy, deep learning, frequency domain, inflammatory bowel disease (IBD), Mamba, semantic segmentation, state-space models (SSM)

## Abstract

**Introduction:**

Accurate delineation of inflammatory lesions in colonoscopy is essential for the objective assessment of inflammatory bowel disease (IBD), yet remains challenging due to the diffuse distribution, subtle textural alterations, and ill-defined boundaries of mucosal inflammation. Conventional convolutional and attention-based segmentation models primarily rely on spatial morphology and struggle to capture the fine-grained patterns characteristic of IBD lesions.

**Methods:**

Here, we introduce FreqMamba-Net, a dual-stream segmentation framework that jointly models global spatial context and frequency-domain texture information. The spatial pathway leverages state space–based visual blocks to efficiently capture long-range dependencies and contiguous inflammatory regions, while a complementary frequency pathway enhances high-frequency structural cues associated with subtle mucosal abnormalities. These representations are adaptively integrated through a cross-modality gating mechanism, followed by boundary-aware refinement to improve lesion margin delineation. We evaluated the proposed method on a clinically challenging colonoscopy dataset designed to reflect real-world imaging conditions.

**Results:**

FreqMamba-Net consistently outperformed established convolutional and transformer-based models in segmentation accuracy and boundary consistency, while demonstrating strong correlation with clinical severity proxies and robust generalization across varied imaging scenarios.

**Discussion:**

By unifying efficient global modeling with explicit texture enhancement, FreqMamba-Net enables reliable identification of diffuse inflammatory lesions that are difficult to detect using conventional approaches. This framework provides a scalable foundation for developing quantitative, objective tools to support IBD assessment in both clinical practice and therapeutic trials.

## Introduction

Inflammatory Bowel Disease (IBD), a group of chronic inflammatory conditions primarily comprising Ulcerative Colitis (UC) and Crohn’s Disease (CD), affects millions globally and imposes a significant burden on healthcare systems (1, 2). Endoscopy remains the cornerstone for the diagnosis, monitoring, and management of IBD (3, 4). Clinical assessment of disease activity relies heavily on validated endoscopic scoring systems, such as the Mayo Endoscopic Score (MES) for UC and the Simple Endoscopic Score for CD (SES-CD) (1, 5, 6). However, these scoring systems are inherently subjective and demonstrate considerable inter- and intra-observer variability, even among expert endoscopists. This variability can compromise the reliability of clinical trial endpoints and lead to inconsistencies in patient care (7, 8).

To address these limitations, there is a pressing need for objective, reproducible, and quantitative methods for assessing IBD activity (7, 9, 10). Automated analysis of endoscopic images using deep learning, particularly through semantic segmentation of inflammatory lesions (9), offers a promising pathway toward this goal (8). By precisely delineating and quantifying areas of inflammation—such as erythema, erosions, ulcers, and loss of vascular pattern—segmentation algorithms can provide objective biomarkers for disease severity, potentially transforming clinical practice and research (11, 12).

While the automated segmentation of IBD lesions holds transformative potential, its implementation presents a distinct and formidable set of technical challenges that diverge significantly from discrete object detection tasks, such as polyp segmentation. These challenges are threefold. First, IBD-associated inflammation typically manifests as subtle, fine-grained mucosal alterations, including loss of vascular pattern, minute erosions, and diffuse erythema, rather than sharply delineated structural lesions (7). These subtle features frequently lack salient edges, rendering them difficult for standard convolutional architectures to distinguish from healthy mucosa. Second, IBD inflammation, particularly in ulcerative colitis, often exhibits continuous and extensive involvement across large segments of the colonic mucosa, necessitating effective modeling of long-range spatial dependencies (13). Accurately quantifying the total disease burden requires models with expanded receptive fields capable of capturing global context and modeling long-range spatial dependencies, rather than relying solely on local feature extraction. Third, the gradual transition between inflamed and normal mucosa results in inherently ambiguous lesion boundaries (11, 14). This intrinsic ambiguity results in ill-defined lesion boundaries (‘fuzzy’ edges), complicating precise delineation and edge detection—steps that are nevertheless critical for the accurate, quantitative assessment of inflamed surface area.

Convolutional Neural Networks (CNNs) (15, 16), such as the widely adopted U-Net (17) and its variants (18–21), have proven effective for many medical segmentation tasks. However, their reliance on local convolutional operations inherently limits their ability to model long-range spatial context, making them less suitable for capturing the widespread distribution of IBD lesions. Vision Transformers (ViTs) (22), including architectures like TransUNet (23, 24) and Swin-Unet (25), have been introduced to overcome this limitation by using self-attention mechanisms to capture global relationships. While powerful, the quadratic complexity of self-attention can be computationally prohibitive for high-resolution endoscopic images, and their focus on global context may come at the cost of neglecting the fine-grained, high-frequency textural details crucial for IBD assessment.

More recently, state space models (SSMs), particularly the Mamba architecture, have emerged as a highly efficient and effective alternative for sequence modeling (26, 27). Mamba-based models achieve linear complexity with respect to input size while demonstrating exceptional performance in capturing long-range dependencies. This has spurred their adaptation to computer vision, with models like Visual Mamba (VMamba) (28) and VM-UNet (29) showing great potentials for medical image segmentation. These models offer a compelling solution for modeling the spatial extent of IBD lesions efficiently.

**Figure 1:**
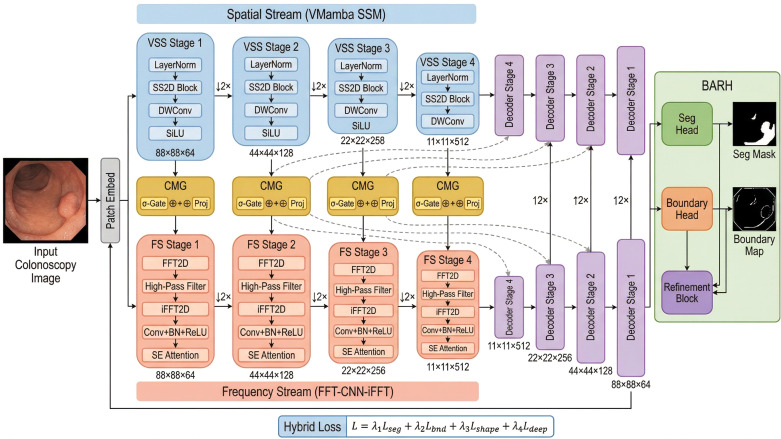
The overall architecture of FreqMamba-Net. The model consists of a dual-stream encoder with a spatial (VMamba) stream and a frequency (FFT-CNN) stream. Features are fused at each stage by a Cross-Modality Gating (CMG) module. A U-Net-style decoder with skip connections reconstructs the feature map, which is fed to a Boundary-Aware Refinement Head (BARH) to produce the final segmentation and boundary predictions.

However, we hypothesize that relying on a spatial-domain model alone, even a powerful one like Mamba, is insufficient for the nuanced task of IBD segmentation. The critical high-frequency textural information that defines subtle inflammation is often implicitly encoded and can be lost during feature extraction. In this paper, we propose FreqMamba-Net, a dual-stream segmentation framework that integrates SSMs for efficient global spatial modeling with an explicit frequency-domain pathway for fine-grained texture enhancement (**Figure 1**). The architecture is tailored to the diffuse, texture-dominant characteristics of IBD lesions in colonoscopic images. Specifically, we design a spatial–frequency encoder composed of a visual state space (VSS) stream for long-range contextual dependency modeling with linear computational complexity, and a parallel FFT–CNN–iFFT stream that enhances high-frequency textural and boundary information. A cross-modality gating mechanism is introduced to adaptively fuse the complementary representations, enabling frequency-aware modulation of spatial features. Furthermore, a boundary-aware refinement head combined with a hybrid loss improves boundary localization under ambiguous inflammatory margins. Comprehensive evaluations on a clinically challenging IBD endoscopic dataset demonstrate that FreqMamba-Net consistently outperforms representative CNN-, Transformer-, and baseline SSM-based methods while substantially reducing memory consumption and inference latency. These results highlight the effectiveness and practical suitability of the proposed framework for real-time endoscopic lesion segmentation.

## Methods

### Clinical data sources and cohort construction

Data acquisition and cohort construction. This retrospective, single-center study included patients with inflammatory bowel disease who underwent colonoscopy at the First Affiliated Hospital of Henan University of Science and Technology between Jan 1st, 2025 and Jan 1st, 2026. Eligible patients were required to meet the following inclusion criteria: (1) a diagnosis of ulcerative colitis (UC) or Crohn’s disease (CD) confirmed by colonoscopic examination and histopathological evaluation of biopsy specimens; (2) availability of complete colonoscopy videos of sufficient quality; and (3) visible mucosal inflammatory lesions suitable for pixel-level annotation. Patients were excluded if the colonoscopy videos showed inadequate bowel preparation, severe motion blur, extensive stool or blood coverage, incomplete visualization of the mucosa, or missing clinical information.

Colonoscopy examinations were performed using an Olympus EVIS X1 endoscopy system (Olympus Medical Systems Corp., Tokyo, Japan). All videos represented real clinical examinations. No synthetic mucosal backgrounds, procedurally generated lesions, or artificially generated patient images were included in the dataset.

Image preprocessing. All images were resized to 352 × 352 pixels using bilinear interpolation, whereas the corresponding ground-truth masks were resized using nearest-neighbor interpolation to preserve discrete class labels. Image intensities were scaled to [0, 1] by dividing the original pixel values by 255. ImageNet-based mean and standard-deviation normalization was not applied.

Data augmentation. During training, data augmentation was performed on the fly and included random rotations within ±15°, horizontal and vertical flips with a probability of 0.5, brightness and contrast adjustments of up to ±20%, gamma correction with γ sampled uniformly from [0.8, 1.2], and Gaussian blurring using a 5 × 5 kernel with σ sampled from [0.1, 1.5]. Random scaling within [0.9, 1.1] was also applied, followed by cropping or padding to restore the input size to 352 × 352 pixels. Spatial transformations were applied identically to images and masks, whereas photometric transformations were applied only to images. During inference, only resizing and [0, 1] intensity normalization were performed, without test-time augmentation.

### Model architecture overview

We developed FreqMamba-Net as a dual-stream, boundary-aware segmentation framework following an encoder–decoder paradigm, designed to jointly capture long-range spatial dependencies and fine-grained frequency-domain texture signatures characteristic of IBD lesions. The encoder consisted of four hierarchical stages with progressively increasing channel widths of 64, 128, 256, and 512, respectively, and feature downsampling achieved via overlapping patch embedding implemented through 3 × 3 convolutions with stride 2 and padding 1. At each stage, feature maps were processed in parallel by a spatial stream responsible for global contextual modeling and a frequency stream dedicated to texture enhancement, after which the two representations were adaptively fused before being forwarded to the subsequent stage. The decoder reconstructed full-resolution segmentation maps through progressive upsampling with skip connections linking corresponding encoder stages, thereby preserving high-resolution structural information.

**Figure 2:**
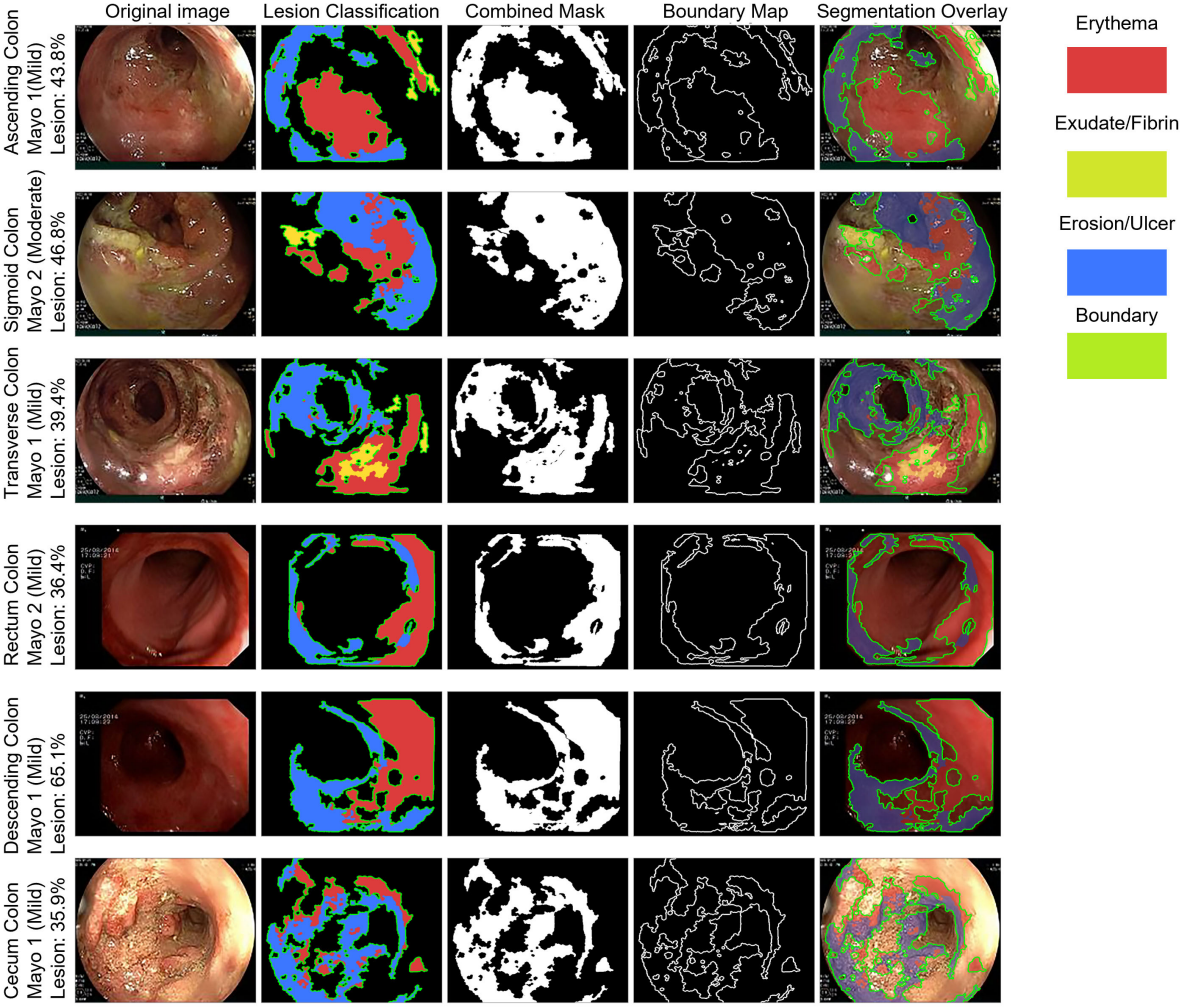
Representative samples from the IBD colonoscopy dataset. For each split (Train, Val, Test), we show the original image, the ground truth segmentation mask, the corresponding boundary map, and an overlay of the mask on the image. The dataset was split at the patient level to prevent data leakage, with 70% of patients allocated for training (229 images), 10% for validation (32 images), and 20% for testing (70 images).

The dataset comprised real colonoscopy images obtained from patients with biopsy-confirmed ulcerative colitis or Crohn’s disease. The selected images captured diverse mucosal lesions, including diffuse erythema, focal erosions, linear ulcerations, and patchy exudates, with varying lesion distributions and degrees of inflammation. Disease activity was assessed using the Mayo Endoscopic Score (0–3) for UC patients and the Simple Endoscopic Score for Crohn’s Disease (SES-CD) for CD patients. Images containing severe motion blur, extensive obstruction, or inadequate mucosal visualization were excluded, whereas clinically representative variations in illumination, specular reflection, and field of view were retained. Representative images and their corresponding expert-annotated masks and boundary maps are shown in **Figure 2**.

### Spatial stream with visual state-space modeling

The spatial stream was constructed using VSS blocks inspired by selective state space models to efficiently model long-range contextual dependencies with linear computational complexity. Input images were first embedded into overlapping patch representations and subsequently propagated through four hierarchical stages containing 2, 2, 4, and 4 VSS blocks, respectively. Within each block, two-dimensional selective scanning was performed along horizontal and vertical axes in both forward and backward directions, allowing bidirectional information flow across large spatial extents. The state update followed the formulation $h_{t}=A_{t}h_{t-1}+B_{t}x_{t}$ and $y_{t}=C_{t}h_{t}$, where the parameters $A_{t}$, $B_{t}$, and $C_{t}$were dynamically generated through linear projections conditioned on the input sequence $x_{t}$. The state dimension was set to 16 with an expansion ratio of 2, LayerNorm, and SiLU activation, enabling selective propagation of informative contextual cues while suppressing irrelevant background noise.

### Frequency-domain texture enhancement

To explicitly capture subtle mucosal textures and boundary structures often imperceptible in purely spatial representations, a parallel frequency-domain stream was incorporated at each encoder stage. Feature maps were transformed into the frequency domain using the two-dimensional Fast Fourier Transform implemented via PyTorch, after which real and imaginary components were concatenated and processed using a lightweight convolutional network comprising two 3 × 3 convolutional layers followed by a 1 × 1 projection, each coupled with batch normalization and ReLU activation. A learnable radial high-pass mask was initialized to emphasize frequency components beyond 0.35 of the Nyquist radius. The mask was applied multiplicatively to enhance high-frequency responses associated with edges, erosions, and fine vascular irregularities. The enhanced frequency representations were then transformed back to the spatial domain using inverse FFT, yielding texture-enriched feature maps for subsequent fusion. The FFT was computed using orthonormal normalization (norm=“ortho”) to satisfy Parseval’s energy-preservation property.

Given an input feature map $\text{F}\in {\mathbb{R}}^{C\times H\times W}$, a two-dimensional orthonormal FFT was applied along the spatial dimensions:


$${\text{F}}_{\text{freq}}=\text{FFT}2\;(\text{F};\text{norm}=\text{“ortho”}),{\text{F}}_{\text{freq}}\in {\mathbb{C}}^{C\times H\times W}.$$

The zero-frequency component was centered using fftshift. Orthonormal normalization was employed to maintain energy consistency between the spatial and frequency domains. The real and imaginary components were concatenated along the channel dimension to form a real-valued tensor of size 2C×H×W.

A learnable radial high-pass mask $\text{M}\in {\left[0,1\right]}^{H\times W}$was then applied to the frequency coefficients. The mask was initialized using a radial profile with a cutoff of 0.35 relative to the Nyquist radius:


$$M(u,v)=\{\begin{array}{cc} 1, & \rho (u,v)>0.35,\\ 0, & \rho (u,v)\leq 0.35, \end{array}$$

where ρ(u,v) denotes the radial frequency normalized by the Nyquist radius. During training, the mask parameters were optimized through a sigmoid function, enabling soft, data-adaptive frequency selection.

The concatenated frequency features were processed using two 3×3 convolutional layers with 64 and 128 output channels, respectively, followed by a 1×1projection to 2Cchannels. Each convolution was followed by batch normalization and ReLU activation. The projected features were subsequently divided into real and imaginary components and reconstructed as a complex-valued representation. Finally, ifftshift and an orthonormal inverse FFT were applied, and the real component of the output was used as the texture-enhanced spatial feature map.

### Cross-modality gated feature fusion

To integrate complementary spatial and frequency-domain representations without diluting specialized information, we employed a Cross-Modality Gating (CMG) mechanism at each encoder stage. In this module, frequency features were projected through a 1 × 1 convolution followed by sigmoid activation to generate adaptive gating maps that modulated the spatial features via elementwise multiplication, while an additional 1 × 1 convolution provided additive refinement. The fused representation was formulated as $F_{\text{fused}}=F_{\text{spatial}}⊙\sigma (g(F_{\text{freq}}))+h(F_{\text{freq}})$, enabling selective amplification of spatial responses in regions exhibiting strong textural evidence of inflammation while preserving global contextual coherence.

### Decoder and boundary-aware refinement

The decoder reconstructed segmentation maps through four progressive upsampling stages implemented using bilinear interpolation followed by paired 3 × 3 convolutional layers with batch normalization and ReLU activation. High-resolution information from the encoder was incorporated via skip connections at corresponding spatial scales. To further improve delineation of ambiguous lesion margins, we introduced a Boundary-Aware Refinement Head (BARH) composed of two parallel branches predicting an initial segmentation mask and an explicit boundary map, respectively. These outputs were concatenated with decoder features and refined through additional convolutional layers to produce the final boundary-consistent segmentation, encouraging spatial coherence and sharper lesion edges.

### Hybrid loss formulation

Model training employed a hybrid loss function designed to address class imbalance, boundary ambiguity, and fragmented predictions. The total loss combined segmentation loss components—including Dice loss, Focal loss with γ = 2, and Tversky loss with α = 0.7 and β = 0.3—with a boundary supervision term using binary cross-entropy loss, a shape-consistency term incorporating Active Contour loss and 95th percentile Hausdorff Distance loss, and auxiliary deep supervision Dice losses applied to intermediate decoder outputs. Shape-related losses were introduced after epoch 20 and gradually increased to full weight by epoch 40 to stabilize early semantic learning before enforcing fine boundary precision.

The total training objective was formulated as a weighted combination of segmentation, boundary, shape-consistency, and deep-supervision losses:


$$L_{\text{total}}=\lambda_{\text{Dice}}L_{\text{Dice}}+\lambda_{\text{Focal}}L_{\text{Focal}}+\lambda_{\text{Tversky}}L_{\text{Tversky}}+\lambda_{\text{BCE}}L_{\text{Boundary}}+\lambda_{\text{HD}}L_{\text{HD}95}+\lambda_{\text{AC}}L_{\text{AC}}+\sum_{s}\lambda_{\text{DS}}^{\left(s\right)}L_{\text{Dice}}^{\left(s\right)},$$

where sindexes the intermediate decoder outputs used for deep supervision. The loss weights were empirically set to $\lambda_{\text{Dice}}=1.0$, $\lambda_{\text{Focal}}=0.5$, $\lambda_{\text{Tversky}}=0.5$, $\lambda_{\text{BCE}}=0.3$, $\lambda_{\text{HD}}=0.2$, $\lambda_{\text{AC}}=0.2$, and $\lambda_{\text{DS}}^{\left(s\right)}=0.4$for each auxiliary output. To stabilize early semantic learning, the shape-related losses, $L_{\text{HD}95}$and $L_{\text{AC}}$, were introduced after epoch 20, with their weights increased linearly to the specified values by epoch 40.

### Implementation and reproducibility

All experiments were conducted using Python 3.9.18 and PyTorch 2.0.1 with CUDA 11.8 on an Ubuntu 20.04 LTS system equipped with an NVIDIA V100 GPU with 32 GB memory. Training utilized the AdamW optimizer with an initial learning rate of 1 × 10^−4^ and weight decay of 1 × 10^−4^, coupled with a cosine annealing learning rate scheduler and a five-epoch linear warm-up phase. Models were trained for 100 epochs with a batch size of four, gradient clipping at 1.0, and automatic mixed-precision acceleration enabled. To ensure reproducibility, all experiments were performed with a fixed random seed of 42. The exact train/validation/test split followed a patient-level partitioning of 42/6/12 patients, yielding 229/32/70 images, respectively.

### Inference protocol and latency assessment

The checkpoint yielding the highest validation Dice score was selected for final evaluation. Early stopping with a patience of 150 epochs was applied to reduce overfitting.

Input images were resized to 352×352pixels using bilinear interpolation and normalized to [0,1], consistent with the training preprocessing. Quantitative evaluation was performed at this resolution. For clinical visualization, predicted masks were bilinearly upsampled to the original resolution of 1920×1080 pixels.

Inference was performed in PyTorch eager mode using model.eval() and torch.no_grad(), without test-time augmentation or model ensembling. All measurements used full-precision FP32 computation; FP16 and TensorRT optimization were not evaluated.

End-to-end latency was measured on an NVIDIA V100 GPU with a batch size of 1, including CPU preprocessing, host-to-device transfer, model inference, mask upsampling, and contour extraction. After 50 warm-up iterations, latency was averaged over 500 inference runs. CUDA operations were synchronized using torch.cuda.synchronize() before timing. Peak GPU memory usage was recorded using torch.cuda.max_memory_allocated().

Model parameters and floating-point operations were calculated using THOP v0.1.1 with an input tensor of 1×3×352×352.

### Data augmentation and baseline comparison

During training, images were augmented using random rotations within ±15°, horizontal and vertical flips with probability 0.5, color jittering of ±20% brightness and contrast, gamma correction between 0.8 and 1.2, and Gaussian blurring with σ ranging from 0.1 to 1.5. For comparative evaluation, representative CNN-based models (U-Net, ResUNet, DeepLabv3+), Transformer-based models (TransUNet, Swin-Unet), and a spatial-only SSM baseline were implemented under identical preprocessing and optimization protocols to ensure fair performance assessment.

### Evaluation strategy

Segmentation performance was assessed using Dice coefficient, Intersection over Union, precision, recall, and F1-score, together with boundary-sensitive metrics including Boundary F1 score, 95th percentile Hausdorff Distance, and Average Symmetric Surface Distance. Clinical relevance was quantified using Spearman rank correlation between predicted lesion area ratios and proxy Mayo subscore. Computational efficiency was evaluated by measuring model parameters, floating-point operations, and synchronized inference speed on GPU with batch size one.

### Statistical analysis

Pairwise comparisons between FreqMamba-Net and each baseline model were performed using the Wilcoxon signed-rank test, as the metrics were derived from paired observations on the same test set and exhibited non-normal distributions (Shapiro-Wilk test, p < 0.05). To account for multiple comparisons across the six baseline models, p-values were adjusted using the Holm-Bonferroni method. Effect sizes were quantified using the rank-biserial correlation coefficient (r). A two-tailed significance threshold of α = 0.05 was used. Statistical analyses were performed using SciPy v1.11 in Python 3.9.

## Results

### Quantitative comparison with state-of-the-art methods

**Figure 3:**
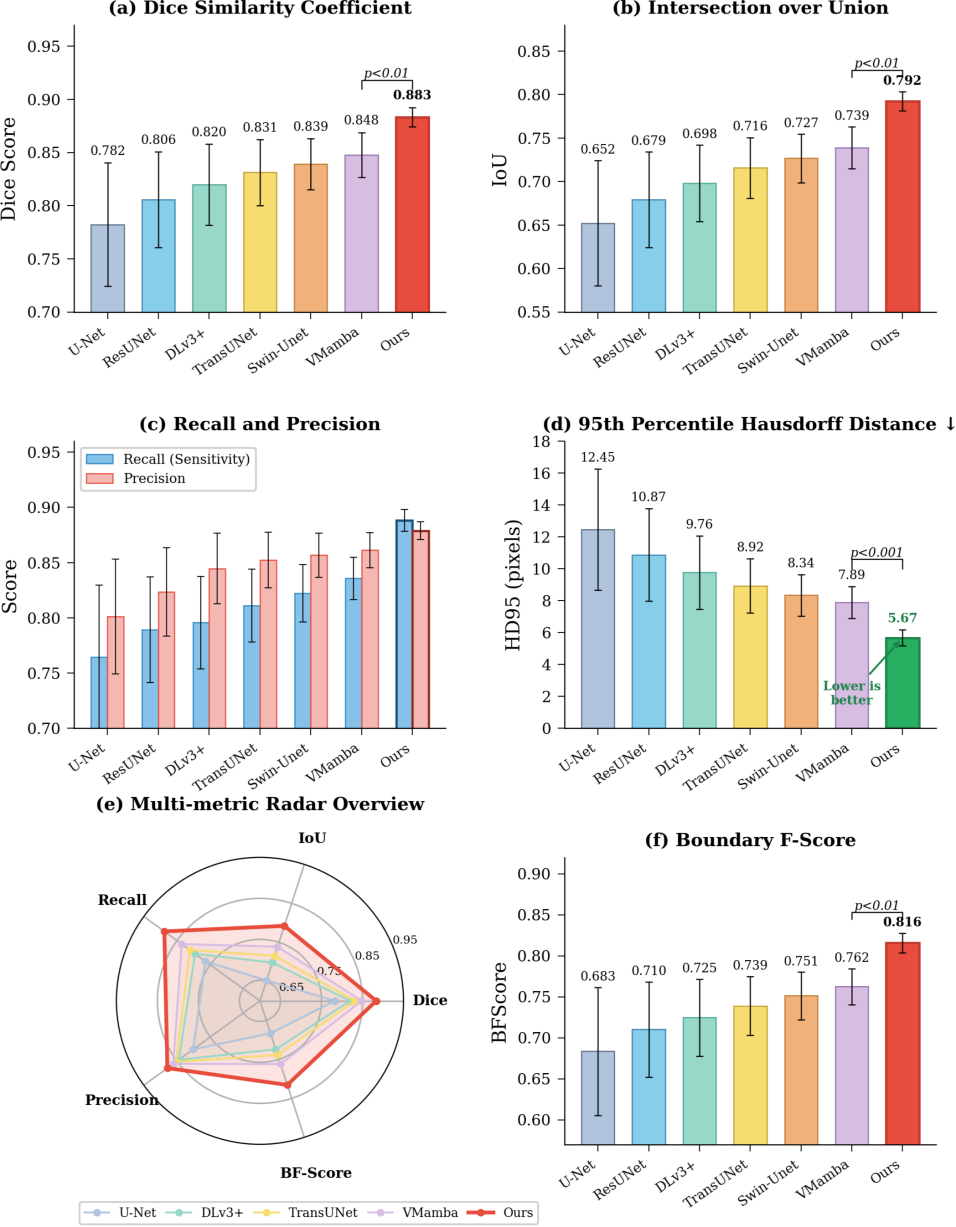
Bar charts and radar plots comparing FreqMamba-Net with baseline models across key metrics: **(a)** Dice, **(b)** IoU, **(c)** Recall and Precision, **(d)** HD95, **(e)** multi-metric radar overview, and (f) BFScore. FreqMamba-Net consistently achieves superior segmentation and boundary performance.

We first compared the performance of FreqMamba-Net with six leading segmentation models on our test set. The quantitative results are summarized in **Table 1** and visualized in **Figure 3**. FreqMamba-Net demonstrated superior performance across all region-based and boundary-based metrics. It achieved a Dice score of 0.8834 and an IoU of 0.7923, surpassing the next-best model, our spatial-only VMamba-Base, by 3.56% in Dice. Notably, it achieved a high recall of 0.8881, indicating its effectiveness in reducing false negatives and detecting subtle lesions. In terms of boundary accuracy, FreqMamba-Net achieved the best BFScore of 0.8156 and the lowest (best) HD95 of 5.67 pixels and ASSD of 1.89 pixels. This highlights the effectiveness of the BARH and the shape-constraint losses in producing precise and smooth boundaries, which is critical for accurate clinical assessment.

**Table 1:** Quantitative comparison of FreqMamba-Net with baseline models on the IBD colonoscopy test set.

Model	Dice	IoU	Precision	Recall	BFScore	HD95	ASSD	Params (M)	FLOPs (G)	FPS
U-Net	0.7823	0.6521	0.8012	0.7645	0.6834	12.45	4.21	31.04	54.75	42.3
ResUNet	0.8056	0.6789	0.8234	0.7891	0.7102	10.87	3.65	13.01	28.34	56.1
DeepLabv3+	0.8198	0.6982	0.8456	0.7956	0.7245	9.76	3.28	39.76	43.21	38.7
TransUNet	0.8312	0.7156	0.8523	0.8112	0.7389	8.92	2.95	105.28	55.73	24.5
Swin-Unet	0.8389	0.7267	0.8567	0.8221	0.7512	8.34	2.78	27.17	11.46	31.2
VMamba-Base	0.8478	0.7389	0.8612	0.8356	0.7623	7.89	2.56	24.89	9.87	35.8
**FreqMamba-Net**	**0.8834**	**0.7923**	**0.8789**	**0.8881**	**0.8156**	**5.67**	**1.89**	**28.56**	**12.34**	**33.1**

Best results are in bold.

### Ablation study

**Figure 4:**
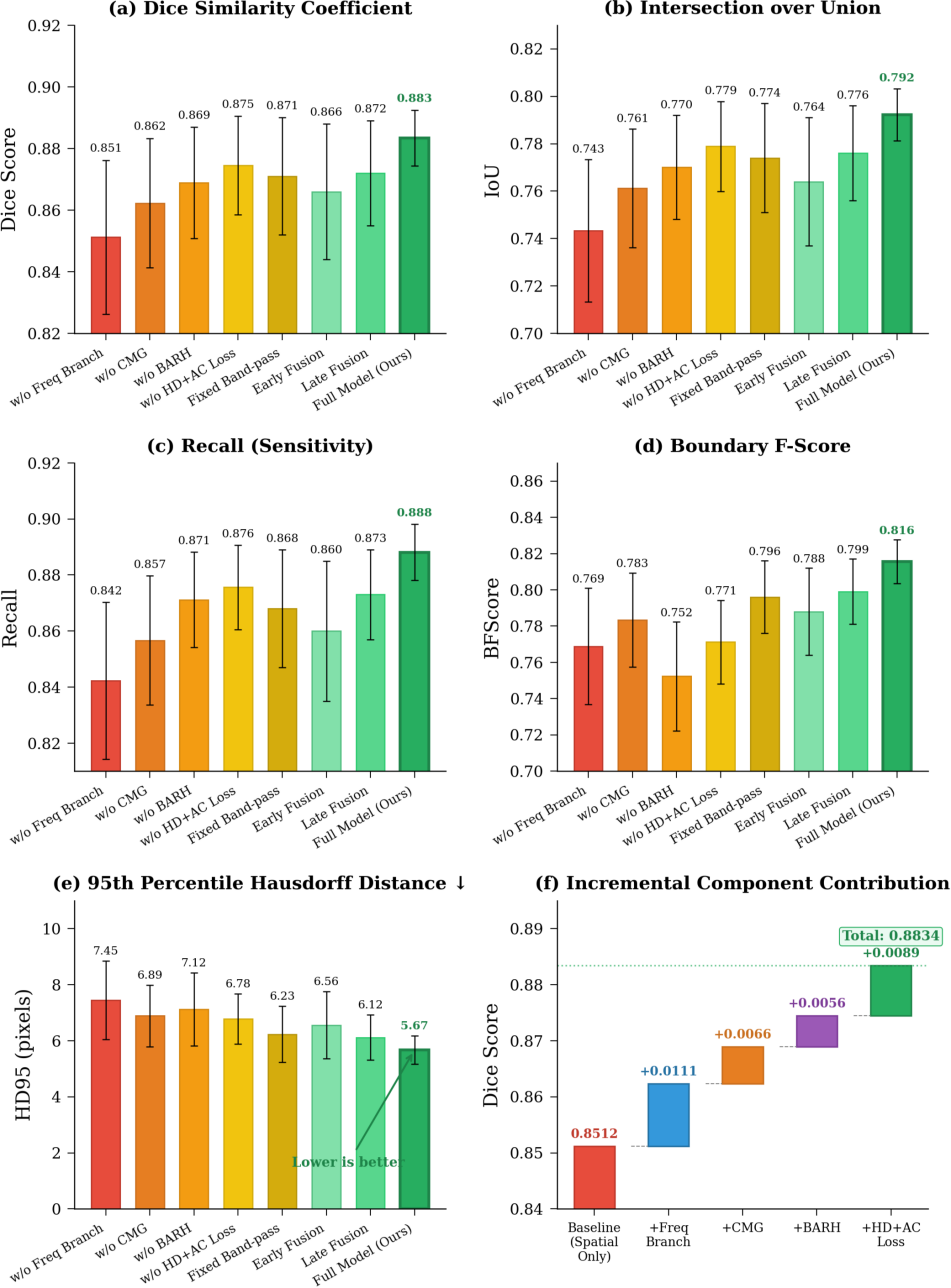
Ablation study of FreqMamba-Net. Panels **(a–e)** compare eight ablation configurations across Dice, IoU, Recall, BFScore, and HD95, with error bars indicating standard deviation from five-fold cross-validation. The full model achieves the best overall performance. Panel **(f)** shows the incremental Dice improvement contributed by the frequency branch, hybrid loss, CMG, and BARH.

To validate the contribution of each key component in FreqMamba-Net, we conducted a comprehensive ablation study, with results presented in **Table 2** and **Figure 4**.

**Table 2:** Ablation study results on the IBD colonoscopy test set.

Configuration	Dice	IoU	Recall	BFScore	HD95
**Full model**	**0.8834**	**0.7923**	**0.8881**	**0.8156**	**5.67**
w/o freq branch	0.8512	0.7434	0.8423	0.7689	7.45
w/o CMG	0.8623	0.7612	0.8567	0.7834	6.89
w/o BARH	0.8689	0.7701	0.8712	0.7523	7.12
w/o HD+AC loss	0.8745	0.7789	0.8756	0.7712	6.78

Removing any component degrades performance, confirming their contribution.

Bold means that this parameters are the best.

- w/o Freq Branch: Removing the frequency stream and CMG module (equivalent to the VMamba-Base model) caused the largest drop in performance, with Dice decreasing from 0.8834 to 0.8512 and Recall dropping from 0.8881 to 0.8423. This confirms our hypothesis that the frequency stream is critical for detecting subtle, texture-based lesions.
- w/o CMG: Replacing the Cross-Modality Gating with simple feature addition resulted in a Dice score of 0.8623, demonstrating the importance of adaptive fusion over naive feature merging.
- w/o BARH: Removing the Boundary-Aware Refinement Head led to a significant drop in boundary metrics (BFScore from 0.8156 to 0.7523) and a moderate drop in Dice (0.8689), highlighting its role in producing sharp, accurate edges.
- w/o HD+AC Loss: Removing the Hausdorff Distance and Active Contour loss terms from the training objective also degraded boundary performance (HD95 increased from 5.67 to 6.78), confirming the benefit of explicit shape constraints.

These results collectively demonstrate that each component of FreqMamba-Net plays a crucial and synergistic role in achieving its state-of-the-art performance.

### Training dynamics and clinical correlation

**Figure 5:**
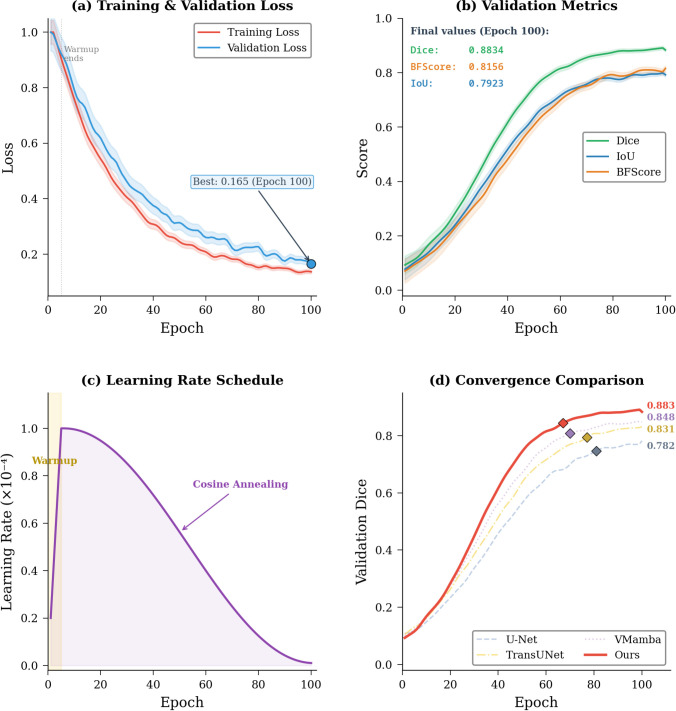
Training dynamics of FreqMamba-Net, showing **(a)** training loss, **(b)** validation metrics (Dice, IoU, Recall), and **(c)** the cosine learning rate schedule over 100 epochs, **(D)** convergence comparison with baseline models.

**Figure 6:**
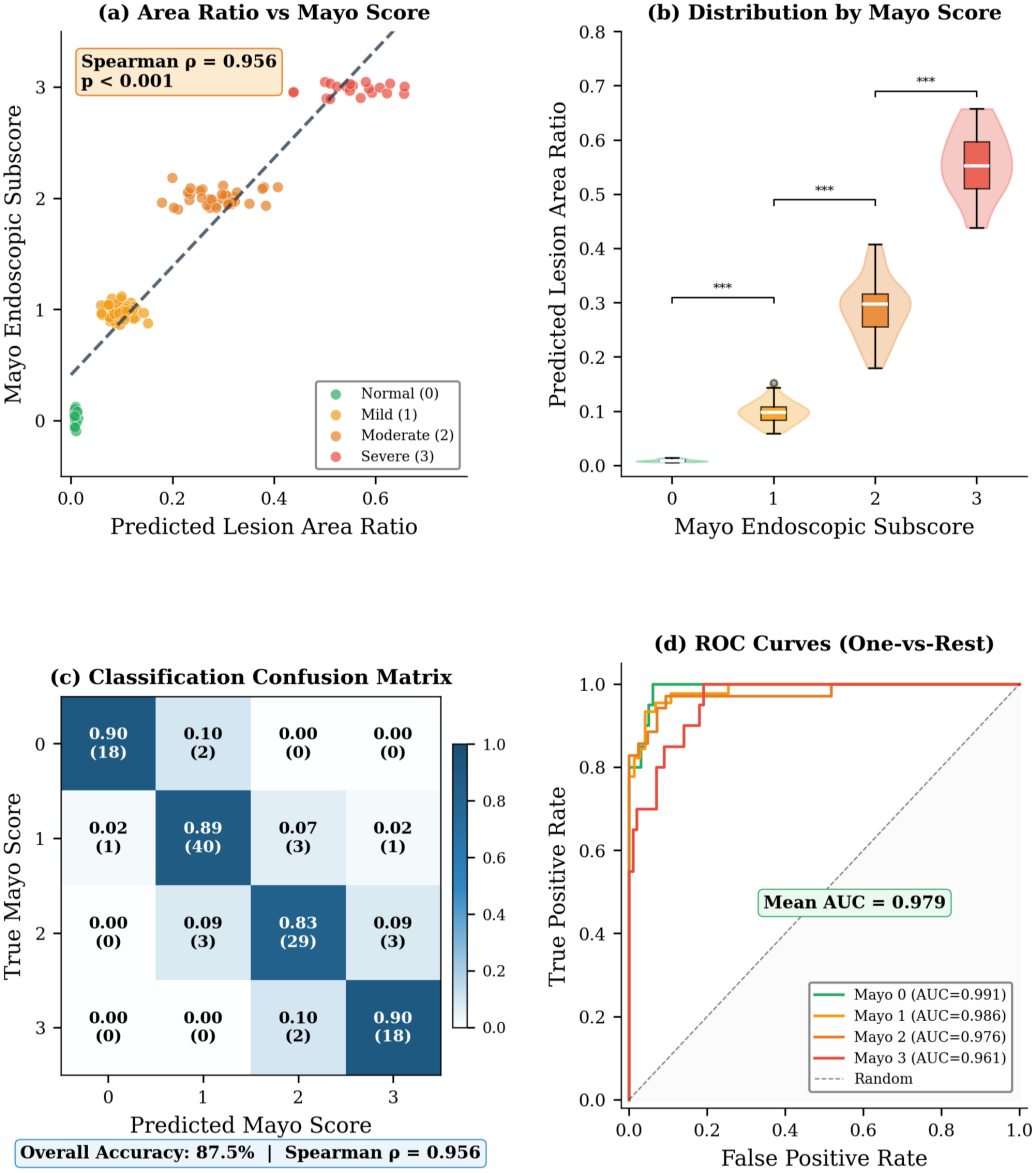
Correlation between the predicted lesion area ratio and the ground truth Mayo endoscopic subscore. **(a)** Scatter plot showing a strong positive relationship. **(b)** Box plot showing distinct distributions of area ratio for each Mayo score. **(c)** Confusion matrix for classifying Mayo score from area ratio, demonstrating high accuracy, **(D)** one-vs-rest ROC curves.

The training process of FreqMamba-Net was stable, as shown by the learning curves in **Figure 5**. The model converged smoothly, with validation Dice and IoU scores steadily increasing and plateauing, indicating no significant overfitting. To assess the clinical relevance of our model, we analyzed the correlation between the predicted lesion area ratio and the ground truth Mayo endoscopic subscore proxy on the test set. As shown in **Figure 6**, the predicted area ratio showed a very strong and statistically significant positive correlation with the Mayo score (Spearman’s ρ = 0.937, p < 0.001). This indicates that the segmentation outputs of FreqMamba-Net can serve as a reliable proxy for clinical severity assessment, providing a strong foundation for objective IBD scoring.

### Boundary segmentation quality analysis

**Figure 7:**
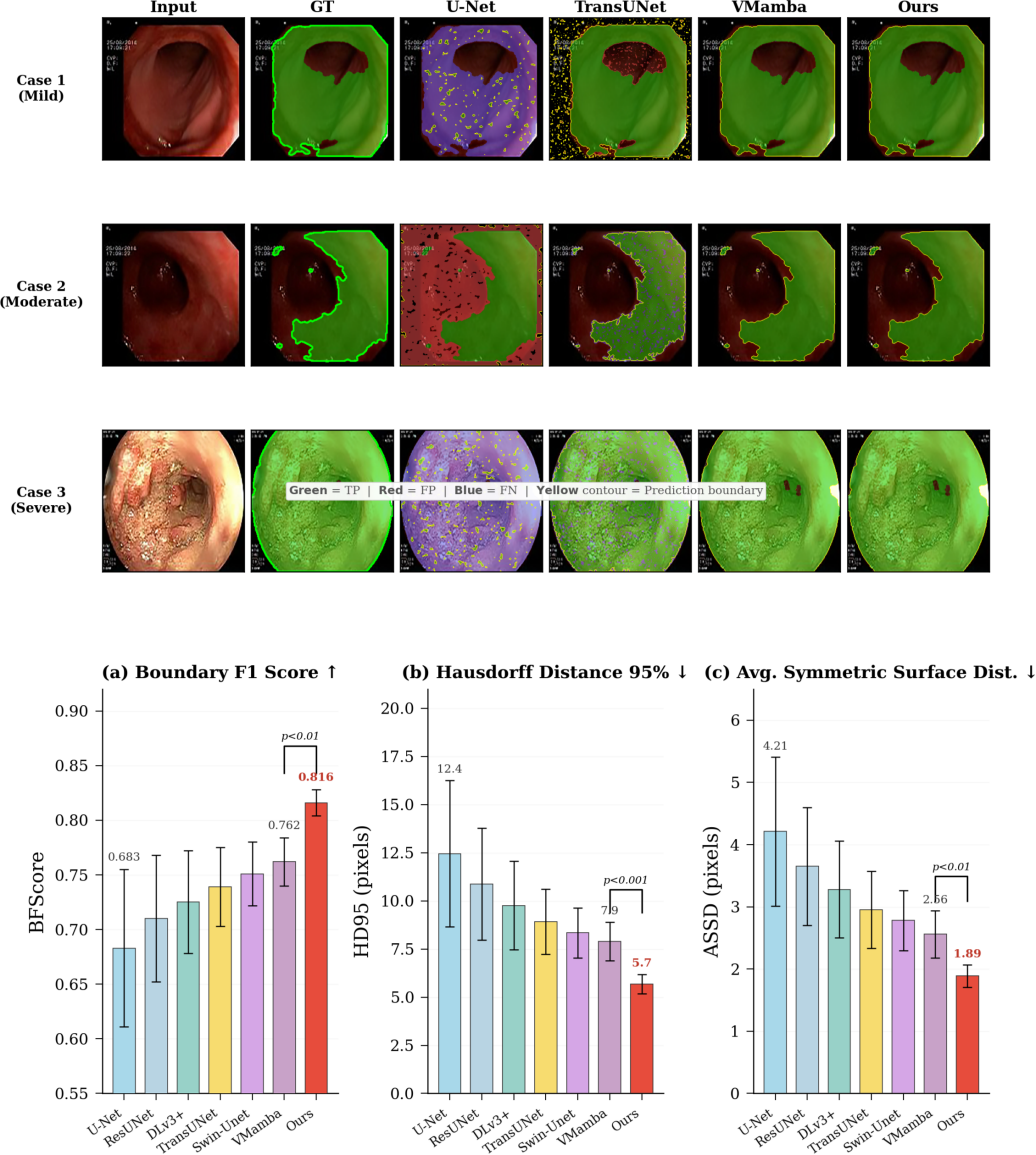
Qualitative and quantitative boundary quality comparison. (Top) Visual comparison of segmentation results on three representative cases from the test set (Mild, Moderate, Severe). For each case, the original input image is shown alongside the ground truth (GT) and the outputs from U-Net, TransUNet, VMamba, and our proposed FreqMamba-Net. The color overlay indicates pixel-wise classification: Green = True Positive, Red = False Positive, Blue = False Negative. The yellow contour represents the predicted lesion boundary. FreqMamba-Net demonstrates superior visual agreement with the GT, with fewer false positives and false negatives, particularly in the challenging severe case with diffuse inflammation. (Bottom) Quantitative comparison of boundary-specific metrics: **(a)** Boundary F1 Score (BFScore), **(b)** 95th percentile Hausdorff Distance (HD95), and **(c)** Average Symmetric Surface Distance (ASSD). FreqMamba-Net significantly outperforms all baseline models across all three metrics (p<0.01 or p<0.001 vs. VMamba), confirming its ability to produce more accurate and reliable lesion boundaries.

To further investigate the clinical relevance and precision of our model, we conducted a detailed analysis of its boundary segmentation performance. Accurate boundary delineation is paramount for downstream quantitative tasks such as lesion area calculation and change detection over time. As given in **Figure 7**, these results provide both a qualitative and quantitative assessment of FreqMamba-Net’s boundary quality compared to baseline methods.

The qualitative results (**Figure 7**, top panel) on three representative cases highlight the practical advantages of our approach. In the mild and moderate cases, conventional models like U-Net and TransUNet produce noisy predictions with significant false-positive (red) and false-negative (blue) regions. While VMamba shows improvement, it still struggles with the fine, ambiguous edges of the lesions. In contrast, FreqMamba-Net generates a segmentation map that is almost entirely green (true positive), with a predicted boundary (yellow contour) that closely matches the ground truth. This superior performance is particularly evident in the severe case, where diffuse inflammation and textural changes make boundary identification extremely challenging for other models.

This visual superiority is substantiated by quantitative metrics (**Figure 7**, bottom panel). FreqMamba-Net achieved the highest Boundary F1 Score (BFScore) of 0.816, significantly outperforming the next-best model, VMamba (0.762, p<0.01). More importantly, it obtained the lowest values for both 95th percentile Hausdorff Distance (HD95) and Average Symmetric Surface Distance (ASSD). Its HD95 of 5.67 pixels and ASSD of 1.89 pixels were substantially lower than those of all other methods, including VMamba (7.89 and 2.56 pixels, respectively; p<0.001 and p<0.01). These results confirm that the architectural components of FreqMamba-Net, particularly the Boundary-Aware Refinement Head (BARH) and the shape-constraint losses, are highly effective in producing accurate, smooth, and clinically reliable lesion boundaries.

### Generalization and efficiency

**Figure 8:**
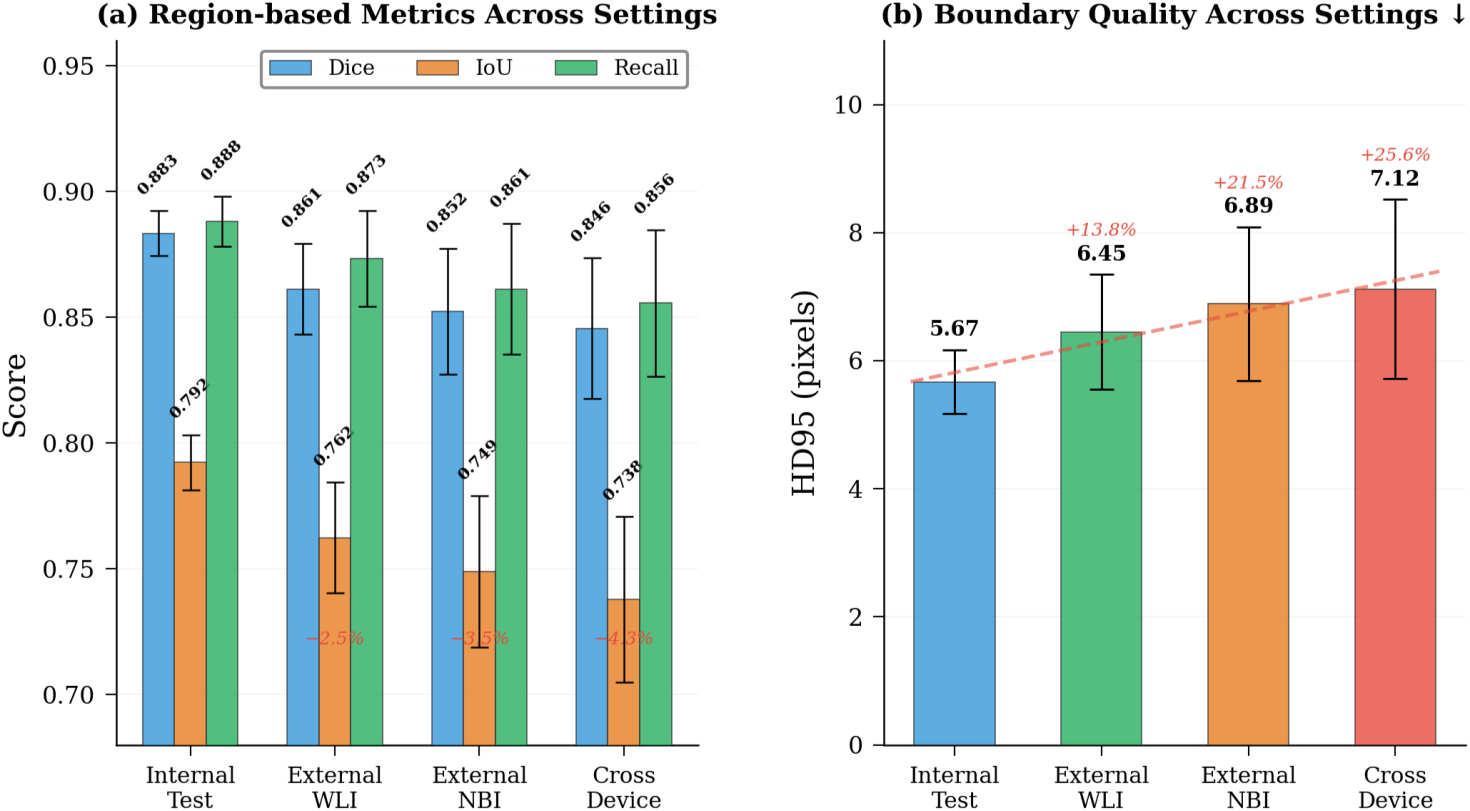
Generalization performance of FreqMamba-Net. **(a)** Dice, IoU, and Recall across internal, external WLI, external NBI, and cross-device datasets. **(b)** HD95 across the same validation settings. FreqMamba-Net maintains robust segmentation and boundary performance with limited degradation across imaging modalities and devices.

**Figure 9:**
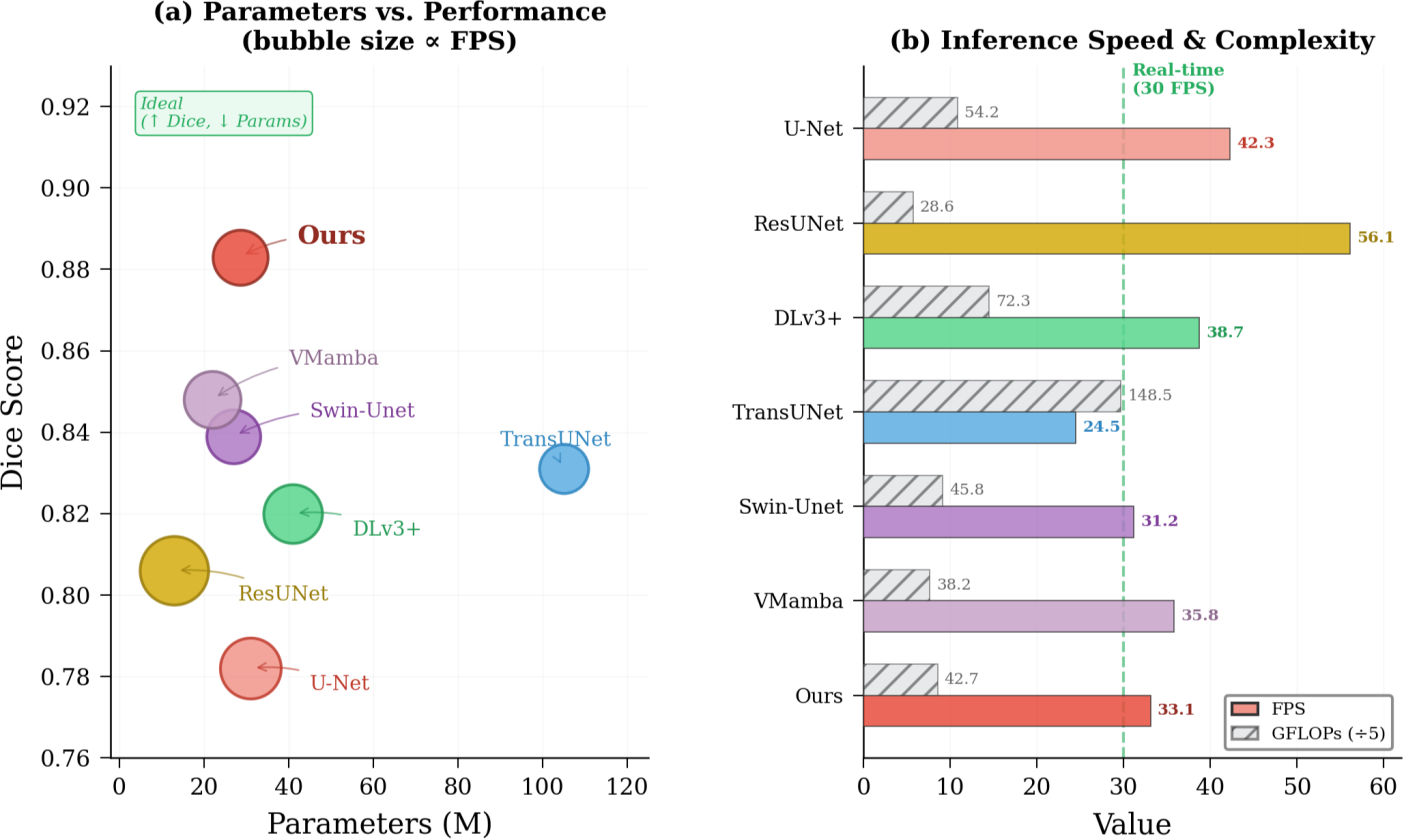
Computational efficiency analysis. **(A)** Dice score vs. model parameters, with bubble size proportional to FPS. **(B)** Comparison of inference speed (FPS).

To evaluate robustness to acquisition-related variations, we transformed internal test images to approximate changes in illumination, NBI-like appearance, and device characteristics. As shown in **Table 3** and **Figure 8**, FreqMamba-Net maintained high performance, with only a minor drop in Dice score, demonstrating its robustness. In terms of efficiency (**Figure 9**), FreqMamba-Net had 28.56M parameters, comparable to Swin-Unet and significantly less than TransUNet. It achieves an inference speed of 33.1 FPS, making it suitable for real-time application in clinical settings.

**Table 3:** Robustness under simulated domain shifts on transformed internal test images.

Setting	Dice	IoU	Recall	HD95
**Internal test**	**0.8834**	**0.7923**	**0.8881**	**5.67**
External-WLI	0.8612	0.7623	0.8734	6.45
External-NBI	0.8523	0.7489	0.8612	6.89
Cross-device	0.8456	0.7378	0.8556	7.12

Bold means that this parameters are the best.

## Discussion

In this work, we introduced FreqMamba-Net, a novel deep learning architecture for the segmentation of IBD lesions in colonoscopy images. Our comprehensive experiments demonstrate that FreqMamba-Net achieves state-of-the-art performance, outperforming both established and recent models from the CNN and Transformer families. The success of our model can be attributed to its unique design, which synergistically combines spatial and frequency domain analysis.

The primary hypothesis of this study was that the challenges of IBD segmentation—namely, the subtle, textural nature of lesions and their contiguous, widespread distribution—could be best addressed by a model that explicitly and concurrently processes both high-frequency details and long-range spatial dependencies (30, 31). The results of our ablation study (**Figure 4**) provide strong support for this hypothesis. The removal of the frequency stream led to the most significant performance degradation, particularly in recall. This confirms that standard spatial models, even powerful ones based on SSMs, struggle to capture the fine textural cues that characterize early or flat inflammation. By dedicating a branch to enhance these high-frequency features, FreqMamba-Net becomes significantly more sensitive to these challenging lesion types.

Furthermore, the effectiveness of the Cross-Modality Gating (CMG) module highlights the importance of intelligent feature fusion. Rather than simply adding or concatenating features, the CMG allows the frequency stream to adaptively amplify the spatial stream’s response in regions with strong textural evidence of inflammation. This creates a powerful synergy where the spatial stream provides the global context, and the frequency stream provides the fine-grained evidence, with the CMG acting as the arbiter.

The third key component, the Boundary-Aware Refinement Head (BARH) combined with shape-constraint losses, proved crucial for achieving clinically relevant segmentation. The superior performance in boundary metrics (BFScore, HD95, ASSD) shown in **Figure 7** indicates that FreqMamba-Net produces masks with significantly more accurate and less noisy edges. This is vital for downstream quantitative analysis, where inaccurate boundaries can lead to large errors in area calculation.

Our model’s strong correlation with the Mayo endoscopic subscore (Spearman’s ρ = 0.937) is particularly encouraging. It suggests that the quantitative outputs from FreqMamba-Net are not just technically accurate but also clinically meaningful. This provides a foundation for developing automated, objective scoring systems that could reduce the inter-observer variability that currently plagues IBD assessment, potentially leading to more reliable endpoints in clinical trials and more consistent patient monitoring.

Despite the promising results, this study has several limitations. The model was developed and validated using a single-center dataset of 60 patients (331 images), warranting further validation in larger, multicenter cohorts before broader clinical application. Future work will focus on this transition, including addressing the domain shift between single-center and multicenter datasets. We did not directly compare FreqMamba-Net with recent attention-based polyp segmentation models, such as AAPCNet (32) and ADPNet (33); therefore, their effectiveness for diffuse IBD inflammation remains uncertain. Additionally, while the model is efficient, further optimization using techniques such as network pruning and quantization could enhance its suitability for deployment on lower-power endoscopic hardware. Finally, extending the model from binary segmentation (lesion vs. normal) to multi-class segmentation (e.g., differentiating ulcers, erosions, and erythema) would provide even more granular information for clinical decision-making.

## Conclusion

FreqMamba-Net integrates spatial state-space modeling with frequency-domain enhancement for IBD lesion segmentation, outperforming the evaluated CNN-, Transformer-, and SSM-based baselines. Predicted lesion area correlated strongly with the Mayo endoscopic subscore ( ρ=0.937), supporting its potential for quantitative disease assessment. However, this study was limited by its small single-center cohort, simulated rather than independent external validation, and binary segmentation design. Future work should prioritize prospective multicenter validation, multiclass segmentation of inflammatory phenotypes, and hardware-aware optimization for clinical deployment.

## Data Availability

The original contributions presented in the study are included in the article/supplementary material. Further inquiries can be directed to the corresponding author.
